# Stressors during the prodromal phase of major depressive episode (CHR-D)

**DOI:** 10.3389/fpsyt.2024.1389370

**Published:** 2024-09-30

**Authors:** Eva Meisenzahl, Frauke Schultze-Lutter, Veronika Stegmüller, Gerd Schulte-Körne, Ellen Greimel, Cosima Klingele, Udo Dannlowski, Tim Hahn, Georg Romer, Marcel Romanos, Lorenz Deserno, Christian Theisen, Milenko Kujovic, Stefan Ruhrmann, Andreas J. Forstner, Natalia Wege

**Affiliations:** ^1^ Department of Psychiatry and Psychotherapy, Medical Faculty, Heinrich-Heine University, Düsseldorf, Germany; ^2^ Department of Child and Adolescent Psychiatry, Psychosomatics and Psychotherapy, University Hospital, Ludwig-Maximilians-Universität (LMU) Munich, Munich, Germany; ^3^ Institute for Translational Psychiatry, University of Muenster, Muenster, Germany; ^4^ Department of Child Adolescence Psychiatry and Psychotherapy, University of Muenster, Muenster, Germany; ^5^ Centre of Mental Health, Department of Child and Adolescent Psychiatry, Psychosomatics and Psychotherapy, University of Wuerzburg, Wuerzburg, Germany; ^6^ Max Planck Institute for Human Cognitive and Brain Sciences, Leipzig, Germany; ^7^ Neuroimaging Center, Technical University of Dresden, Dresden, Germany; ^8^ Department of Psychiatry and Psychotherapy, Faculty of Medicine and University Hospital, University of Cologne, Cologne, Germany; ^9^ Institute of Human Genetics, University of Bonn, School of Medicine and University Hospital Bonn, Bonn, Germany

**Keywords:** major depression, stressors, prodromal phase, CHR-D, early recognition, indicated prevention

## Abstract

**Introduction:**

Early recognition and indicated prevention is a promising approach to decrease the incidence of Major depressive episodes (MDE), targeting the patients during their clinical high-risk state of MDE (CHR-D). The identification of a set of stressors at the CHR-D increases the success of indicated prevention with personalized early interventions. The study evaluated stressors in the early phase of depression, developed on the basis of a patient survey on stressors.

**Methods:**

Sixty-eight inpatients (ICD10: F3x.xx) with a reported high risk state for major depressive episode (CHR-D) were included in the current study. Stressors during CHR-D were retrospectively explored using a semi-structured clinical interview supplemented by open-ended questions. A qualitative explorative content analysis was provided to identify a pattern of stressors during the prodromal phase of the patients, based on the patient’s perspective. A frequency analysis was performed for the evaluation of the prevalence of reported source of stress.

**Results:**

All patients reported stressors in the prodromal phase of depression. Results demonstrates that patients with depressive disorder typically report multiple stressors, with the most common number being four. First, 18 stressors-groups were identified during coding. Interpersonal conflicts and disappointments in close relationships were most frequently reported stressors during the prodromal phase at 44.1%. The second most frequent stressor mentioned was the high qualitative or quantitative demands at work (38.2%). The third frequent source of stress was changes in close relationships and in family relationships (33.8%). Based on the categories of stressors described in the patient survey during the prodromal phase we suggested a model of stressors in CHR-D during the prodromal phase of the MDE.

**Discussion:**

The identification of a set of stressors at the early stage of MDE may increase opportunities for early intervention. In everyday clinical practice, preventive psychiatry needs clinical and adapted instruments for recording stressors in today’s society. This knowledge is necessary in order to develop precisely indicated prevention for depressive disorders.

## Introduction

Depression is the leading cause of disability worldwide, making its prevention a crucial focus for mental health initiatives (WHO). Indicated prevention, which involves early interventions for individuals at high risk of developing depressive disorders, has been shown to be particularly effective (1, 2). Our recent study assessed the occurrence of clinical high-risk states of depression (CHR-D), describing the length and symptoms of the prodromal phase (3). Understanding the complex, multifactorial nature of depression (4) and identifying modifiable environmental risk factors are essential for developing targeted preventive strategies. While the relationship between stress and depression is well-established, there is a critical need to systematically assess specific stressors during the early stages of depression.

The role of stress in the onset of depression is well-established. Various stressors, such as perceived stress (5), environmental factors, academic stress (6), and work-related stress (7, 8), health related factors (9, 10) are known to influence depression risk. However, systematic assessments of stressors during the early stages of depression are limited. Yet specific stressors during the prodromal phase remain under-explored.

Perceived stress, defined as the extent to which situations in one’s life are appraised as stressful, has been extensively studied in relation to depression. The Perceived Stress Scale (PSS) developed by Cohen et al. (5) is a widely used tool to assess this construct. Research consistently shows that higher perceived stress is associated with an increased risk of developing depression (5). Recent reviews support these findings, indicating that perceived stress is a robust predictor of depressive symptoms across populations (11). For example, a meta-analysis by Li et al. (12) examines the association between perceived stress and depression among medical students in China, highlighting the significant impact of stress on mental health. Hammen (13) offers a comprehensive review of various risk factors for depression, including life events, and discusses the mechanisms through which stressors contribute to the onset of depressive disorders. Kendler and Gardner (14) explore how stressful life events and previous depressive episodes predict the development of major depression, emphasizing the role of life stress in depression recurrence.

Environmental factors also play a significant role in depression. A meta-analysis by Roberts et al. (15) found that exposure to green spaces positively affects reducing depression and anxiety symptoms, highlighting the importance of environmental factors in mental health. Lim et al. (16) evaluated the impact of air pollution on depression, finding significant associations between exposure to particulate matter (PM2.5) and an increased risk of depression. This underscores air pollution as a critical environmental stressor contributing to mental health issues. An umbrella review by Cuijpers et al. (17) synthesized findings from various meta-analyses on the impact of climate-related events on mental health, concluding that exposure to extreme climate events is associated with a higher prevalence of depression, anxiety, and PTSD.

Psychosocial factors, such work-related stress, particularly in high-demand, low-control jobs, has been linked to increased depression risk. The Effort-Reward Imbalance (ERI) Model (7), the Job Demand-Control (JDC) Model (18), and the concept of Organizational Justice provide frameworks for understanding these associations. Meta-analyses have shown that high effort-reward imbalance is associated with a significantly higher risk of depression (19). The JDC Model suggests that high job demands coupled with low control are associated with increased depression risk (18). A systematic review and meta-analysis by Theorell et al. (20) confirmed these findings. Organizational Justice, referring to fair treatment in the workplace, is also linked to higher levels of stress and depression (21). A meta-analysis by Ndjaboué et al. (22) found significant links between perceptions of injustice in the workplace and depressive symptoms. Academic stress is another significant contributor to depression, particularly among students. Andrews and Wilding (6) identified academic stress as a key predictor of depressive symptoms in university students, a finding supported by recent meta-analyses (23).

Understanding specific stressors as triggers for depression is crucial. For instance, Piechaczek et al. (24) found that youths with major depression experienced a higher proportion of specific psychosocial stressors compared to their peers. Financial stressors, such as income loss and debt, are strongly associated with an increased risk of depression in adults (25). Individual factors also play a role, with somatic symptoms being associated with depression and predicting its presence in primary care (9). Changes in antidepressant medication were shown to increased risks of relapse and recurrence (10).

Despite extensive research on stress and depression, a gap remains in systematically assessing stressors during the early stages of depression. Most studies focus on stressors after the onset of depressive symptoms, leaving a critical gap in understanding the role of stressors relevant in the prodromal phase. The current study aims to fill this gap by exploring stressors during the prodromal phase of depression from the patient’s perspective. By identifying relevant stressors and their patterns, our research seeks to enhance the predictive power of existing models and inform early intervention strategies. Building on findings from previous studies, our research incorporates a broader range of stressors and individual factors. This approach aims to develop more accurate predictive models for depression, enabling early identification and preventive interventions for individuals at high risk.

The existing tools for assessing stress in mental illnesses such as depression often lack specificity and do not always align with the latest societal changes and demands. Consequently, there is an urgent need to conceptually re-evaluate stressors through patient interviews to ensure that the identified stressors in high-risk patients are effectively incorporated into future indicated prevention strategies for depression. While the broad determinants of depression are established, focusing on the nuances of the prodromal phase could refine early diagnostic tools, making them more personalised. Therefore, the manuscript’s aim is to underline the importance of distinguishing these early stressors. This detailed understanding could contribute significantly to the field of preventive psychiatry by enabling early interventions tailored to the specific stressors that precede clinical depression.

For this aim, we developed a survey to capture stressors based on patient feedback and subsequently constructed a model of socially relevant stressors. The primary objective was to identify the stressors and patterns associated with the clinical high-risk state of depression (CHR-D) using the DEEP-IN Inventory, detailed extensively by Meisenzahl et al. (3). We employed an open-ended methodology, allowing participants to articulate the stressors they encountered during the prodromal phase of their depressive episodes.

Given the methodological challenges in designing a predictive tool for depression, we adopted an exploratory approach in this initial phase of our research. By considering current societal trends and the trajectory of depressive disorders, this research addresses the existing knowledge gap regarding stressors in the prodromal stage from the patient’s perspective. Our primary interest was to identify critical sources or categories of stressors using a qualitative approach that foregrounds the patients’ perspectives during the prodromal phase of depression.

## Methods

### Study sample

The recruitment procedure involved several systematic steps to ensure the inclusion of eligible participants and adherence to ethical standards.

As a first step, an initial screening of patients at the Department of Psychiatry and Psychotherapy at the Ludwig-Maximilian University in Munich was performed by trained clinicians (E.M., V.S.). This screening involved reviewing patient records to identify individuals who met the basic inclusion criteria, which included being between 18 and 65 years old and having a past or current diagnosis of unipolar depression (F32), recurrent depressive disorder (F33), or bipolar disorder (F31) according to ICD-10 criteria (26).

As a next step, potential participants were assessed for specific inclusion and exclusion criteria. Inclusion criteria required patients to be in partial remission with a Beck Depression Inventory (BDI) score of less than 20 and the ability to provide written informed consent. Exclusion criteria were comprehensive, covering insufficient German language skills, comorbid diagnoses of organic, symptomatic psychopathological disorders, schizophrenia and delusional disorders, developmental disorders, acute suicidality, a history of significant craniocerebral trauma, and other medical conditions affecting brain function. Patients who met the inclusion criteria were approached and provided with detailed information about the study. They were informed about the study’s purpose, procedures, potential risks, and benefits. Those who agreed to participate signed a written informed consent form. The study was conducted in accordance with the ethical guidelines set by the Ethics Committee of the Ludwig-Maximilians University (EK524-15).

A total 85 individuals were agreed to participate in the study. After exclusion of patients with exclusion criteria (substance use, significant craniocerebral trauma, neurological diseases affecting the brain), final sample of 68 participants with completed interviews were available for the analysis.

### Assessments

A semi-structured Depression Early Prediction Interview (DEEP-IN) was conducted to explore the prodromal phase, its duration, prodromal symptoms and clinical course of the affective disorder. The average duration of the DEEP-IN interview was 1.5 hours. First, sociodemographic information including gender, age, marital status, education, occupation, income, nationality/migrant status, and native language, was obtained. Furthermore, clinical information on past and present depressive episodes and utilisation of health care services (inpatient and outpatient) was collected. Additionally for the validation of the diagnosis and the course of the disease episodes, the patient’s medical records and doctor’s letters were examined. Examination of a possible prodromal phase of depression was performed using a semi-structured interview supported by the Life-Chart-Method (27), the detailed procedure is described elsewhere (3).

### Index episode

The investigation of possible stressors of the prodromal phase of depression was supported by the life-chart method according to Lyketsos et al. (27) that was found to perform well in depressive patients (28). In a first step, trained clinicians recorded the number of previous MDEs, their onset and duration. Patients were asked to represent their course of disease graphically in a time-lifeline [onset: month/year; end: month/year; (3)]. To improve the validity of recall, the best remembered MDE by patients was identified as the ‘index episode’. In patients with bipolar disorder, the selected ‘index episode’ was required to proceed the first (hypo) manic episode.

### Stressors of the major depressive episode

As a part of the semi-structured DEEP-IN interview, an exploration of the stressors during the prodromal phase that caused subsequently major depressive episode from the patients’ perspective was conducted by a trained interviewer. Patients were asked if there were stressors that during the prodromal phase triggered the later (or index-) major depressive episode. All stressors relevant to depressive episodes were quoted by the interviewer. Multiple nominations were allowed.

### Statistical analysis

First, descriptive statistics of the study variables were provided. As a next step, qualitative content analysis was conducted to uncover patterns, themes, and categories important for the development of the index depressive episode from the patient’s perspective.

### Content analysis

Both qualitative and quantitative analysis techniques were used for the data analysis. A pragmatic approach guided by principles from inductive grounded theory and thematic analysis was used to analyse qualitative data. Before data analysis and coding commencement, some deductive themes were developed based on the stress research (29). These themes were chosen on an *a priori* basis to ensure any sources of stressors were identified and formed the basis of the thematic analysis. Using inductive grounded theory, a descriptive approach (thematic analysis) was applied, whereby answers were coded in order to develop conceptual categories. Statements dealing with similar topics were grouped in a category and refined them into an overarching theme and subthemes. Two independent researchers, EM and NW, to ensure interrater reliability and enhance the validity of the findings, conducted the content analysis. This dual-coding approach follows recommended practices in qualitative research, where independent coding and subsequent consensus discussions are crucial for minimizing bias and verifying the consistency of the data interpretation (29, 30).

Data was collected using semi-structured interviews that allowed participants to recall their experiences freely while ensuring that key topics related to the prodromal phase of depression were explored. Each interview was conducted by trained clinical researchers and lasted approximately 60-90 minutes. These were employed to elicit detailed responses about the stressors experienced by participants, allowing them to share in-depth personal insights into their prodromal symptoms and associated stressors.

Initial Coding: Using a grounded theory approach, initial codes were generated by reading through the records to identify key themes and patterns related to stressors. This inductive coding was performed independently by two researchers to enhance the reliability of the identified themes.

Focused Coding: Codes that appeared frequently or were deemed significant for understanding the prodromal phase were then grouped into larger categories that described broader themes in the data.

Consensus Meetings: Regular meetings were held between the researchers to compare and refine codes and to resolve any discrepancies in coding, thereby ensuring consistency and depth in the analytical process (EM and NW).

Theme Identification: From the focused codes, major themes were developed that captured the essence of the stressors reported by participants. This involved synthesizing the data to form a coherent narrative about common and unique stressors impacting the early stages of depression.

Validation of Themes: Themes were continually revisited and validated against the data throughout the analysis process to ensure they accurately represented the views and experiences of the participants.

Adapted grounded theory techniques were used with inductive theme creation, as numerous combing/coding cycles were conducted. For example, mentions of interactional stress in close relationships and at work were a frequent topic among many of the participants’ statements, subsequently becoming major themes. Additionally, we included themes less frequently mentioned but relevant to the research, such as physical environmental stressors. These stressors can significantly affect certain individual’s mental health, contributing to a nuanced understanding of depression triggers. This holistic approach ensures the study acknowledges the complex and multifactorial nature of depression, capturing a wide spectre of stressors and prevents overlooking potential contributors that may be critical for specific subgroups.

### Frequency matrix analysis

Upon completion of the qualitative analysis, the data was transformed into a frequency matrix for exploratory quantitative analysis. The resulting categories were included into a frequency analysis. Answers were operationalized in variables with two categories (yes/no). Appropriate individual themes were collapsed into two overarching themes: individual factors and environmental factors. A frequency analysis was performed for the evaluation of the prevalence of reported sources of stressors. Prevalence of reported stressor categories was calculated in percent. Pearson’s Chi-square test was used to compare stressors between groups based on gender and course of disease (first depressive episode vs recurrent depression). The results are given as percentage.

The statistical Package for the Social Sciences (IBM SPSS Statistics, version 28.0) was used for the descriptive data analysis of the qualitative data.

## Results

### Description of the study sample

**Table 1** provides an overview of the sample demographics and health-related characteristics. Of the 68 patients surveyed, 38.2% were male, the mean age was 39.36 years. The majority had completed secondary education, 42% were single, while 33.8% lived alone. Additionally, 58.8% were employed, the majority of patients displayed a net income of over 1500 Euro per month. The mean duration of the prodromal phase was 7.88 months. 35.5% of study participants described their first depressive episode as an index episode, as described above, 64.5% of patients described their further depressive episode of the recurrent depression as an index episode.

**Table 1:** Descriptive characteristics of the study sample.

Variables	Total (N=68)	Percent
Age (mean; SD)	39.36	10.10
Sex		
− Male	26	38.2%
− Female	42	61.8%
School education		
− Less than secondary school	17	25%
− Secondary school	51	75%
Marital status		
− Married	17	25.0%
− Committed relationship	15	22.1%
− Single	29	42.6%
− Divorced	7	10.3%
Children		
− Yes	49	72.1%
− No	19	27.9%
Living conditions		
− Living alone	23	33.8%
− Living with a family	35	51.5%
− Shared apartment	10	14.7%
Employment status		
− Employed	40	58.8%
− Unemployed	12	17.7%
− Student	10	14.8%
− Pension	6	8.8%
Net income		
− ≤450	7	10.3%
− 451-850	6	8.8%
− 851-1500	11	16.2%
− 1501-2500	17	25.0%
− 2501-3500	16	23.5%
− > 3500	6	8.8%
− K.A.		
Prodromal phase duration (mean; SD)	7.88	12.50
Index Episode		
− 1	24	35.3%
− 2	15	22.1%
− 3	13	19.1%
− 4	8	11.8%
− 5	4	5.9%
− 6	2	2.9%
− 8	2	2.9%

### Cumulative analysis of stressors

The **Table 2** presents the distribution of stressors that triggered prodromal phase of depressive episodes from the patient’s perspective. The majority of patients reported between two and four stressors. Specifically, 27.9% of patients reported four stressors, which is the highest frequency among all categories. Only a small proportion of patients reported one (2.9%) or eight (1.5%) stressors, indicating that most patients experience multiple stressors.

**Table 2:** Cumulative stressors exposure bevor onset of depressive episode.

Number of Stressors	At least x stressor reported n (%)	Number of stressors reported n (%)	Family & Close Relation-ships	Work & Study	Psycho-pathology	Somatic Health	Environ-mental Factors	Socio-economic
1st. Stressor	68 (100%)	2 (2.9%)	27 (39.1%)	22 (32.3%)	8 (11.6%)	5 (7.2%)	5 (7.2%)	1 (1.4%)
2d. Stressor	66 (97.0%)	9 (13.2%)	25 (36.8%)	18 (26.1%)	13 (18.8%)	6 (8.7%)	4 (5.8%)	-
3d. Stressor	55 (80.9%)	13 (19.1%)	23 (33.3%)	16 (23.2%)	9 (13.2%)	5 (7.2%)	3 (4.3%)	1 (1.4%)
4th. Stressor	41 (60.2%)	19 (27.9%)	13 (18.8%)	10 (14.5%)	12 (17.6%)	2 (2.9%)	4 (5.8%)	-
5th. Stressor	24 (35.2%)	12 (17.6%)	7 (10.1%)	5 (7.2%)	9 (13.0%)	2 (2.9%)	1 (1.4%)	-
6th. Stressor	10 (14.7%)	8 (11.8%)	5 (7.2%)	1 (1.4%)	-	2 (2.9%)	1 (1.4%)	1 (1.4%)
7th. Stressor	3 (4.1%)	4 (5.9%)	1 (1.4%)	1 (1.4%)	-	-	1 (1.4%)	-

Category ‘Family & Close Relationships’ was most frequently reported as the first stressor (39.1%), and decreases gradually to the seventh stressor (1.4%). Second most frequent first stressor (32.3%) was occupational or academic stress and remains a common stressor through the second (26.1%) and third stressors (23.2%), then decreases. Psychopathology was reported by 11.6% as the first stressor and fluctuates slightly, maintaining relevance up to the fifth stressor (13.0%). Somatic Health was initially reported by 7.2% and remains relevant across all stressor levels, with the highest being the second stressor (18.8%). Environmental Factors starts at 7.2% for the first stressor, then decreases and stabilizes at lower percentages. Socio-economic category was reported minimally, starting at 1.4% for the first stressor, and appears sporadically in other levels.

There are notable differences between the first, second, third, and subsequent triggers reported by the source of the stress. Family & Close Relationships consistently appears as the most frequently reported stressor across the first three stressors and remains present in smaller proportions up to the seventh stressor. Work & Study, initially significant, this stressor’s frequency decreases steadily as subsequent stressors are reported. Psychopathology shows a slight increase in the second and fourth stressors, then diminishes. Somatic Health stressors generally maintains a low but consistent presence across most stressor levels. Environmental Factors starts with a small proportion and remains relatively low across all levels. Socio-economic stressors were rarely reported and appears only in the first and third stressors.

### Qualitative content analysis of stressors

**Table 3** displays 18 inductive (emergent) themes that were utilised during coding. Interactional stress in close relationships in terms of interpersonal conflicts and disappointments was the most frequently reported stressor related to the development of a depressive episode, at 44.1%, when considering the sources of stress overall. The second most frequent stressor mentioned was the high qualitative or quantitative demands at work (38.2%). The third frequent source of stress were life vents in sense of changes in close relationships and in family relationships (33.8%), and other psychopathological symptoms (33.8%). Further stressors mentioned by patients were death or illness of a family member or close friend (32.3%), interactional stress due to conflicts and disappointments at work (25.0%), changes at work or school (22.1%), change of living place (17.6%), trauma reactivation) (14.7%), social isolation (13.2%), somatic symptoms (13.2%), psychopharmacological medication or change of medication (10.3%), physical living environment (13.2%), financial problems and insecurity (5.9%), qualitative and quantitative demands in close relationships (2.9), physical working environment (2.9%), social withdrawal (2.9%) and physical functional limitations (2.9%).

**Table 3:** Results of qualitative analysis - frequencies of stressors, relevant for depressive episode.

Stressors	n	%
Family & close relationship: Interactional stress due to conflicts & disappointments	30	44.1
Work & school: qualitative and quantitative demands	26	38.2
Family & close relationship: changes	23	33.8
Mental health: psychopathological symptoms	23	33.8
Family & close relationship: death or illness	22	32.3
Work & school: Interactional stress due to conflicts & disappointments	17	25.0
Work & school: changes	15	22.1
Living environment: change of living place	12	17.6
Mental health: trauma	10	14.7
Social network: social isolation	9	13.2
Physical health: somatic symptoms	9	13.2
Mental health: medication SE or change of medication	7	10.3
Physical living environment: noise. temperature	4	5.9
Socioeconomics: financial problems & insecurity	4	5.9
Family & close relationship: qualitative and quantitative demands	2	2.9
Physical working environment: working conditions	2	2.9
Social network: social withdrawal	2	2.9
Physical health: functional limitations	2	2.9

### Gender differences of stressors

Significant gender differences were found in the stressors related to family and close relationships, work and study, and psychopathology, with women reporting higher exposure to these stressors compared to men (**Table 4**). Other stressors, such as health-related factors, environmental factors, and financial difficulties, did not show significant gender differences.

**Table 4:** Descriptive statistic of the sex differences in stressors exposure (n, %).

Stressors	Men (n, %)	Women (n, %)	Significance Level (χ², df, p-value)
Family and Close Relationships	34 (53.1%)	48 (70.6%)	3.89, 1, p=0.048
Work and Study	26 (40.6%)	39 (57.4%)	4.15, 1, p=0.042
Psychopathology	15 (23.4%)	32 (47.1%)	8.41, 1, p=0.004
Somatic Factors	12 (18.8%)	18 (26.5%)	1.09, 1, p=0.295
Environmental Factors	7 (10.9%)	10 (14.7%)	0.30, 1, p=0.582
Financial Difficulties	7 (10.9%)	7 (10.3%)	0.01, 1, p=0.941

### Conceptual considerations

**Figure 1:**
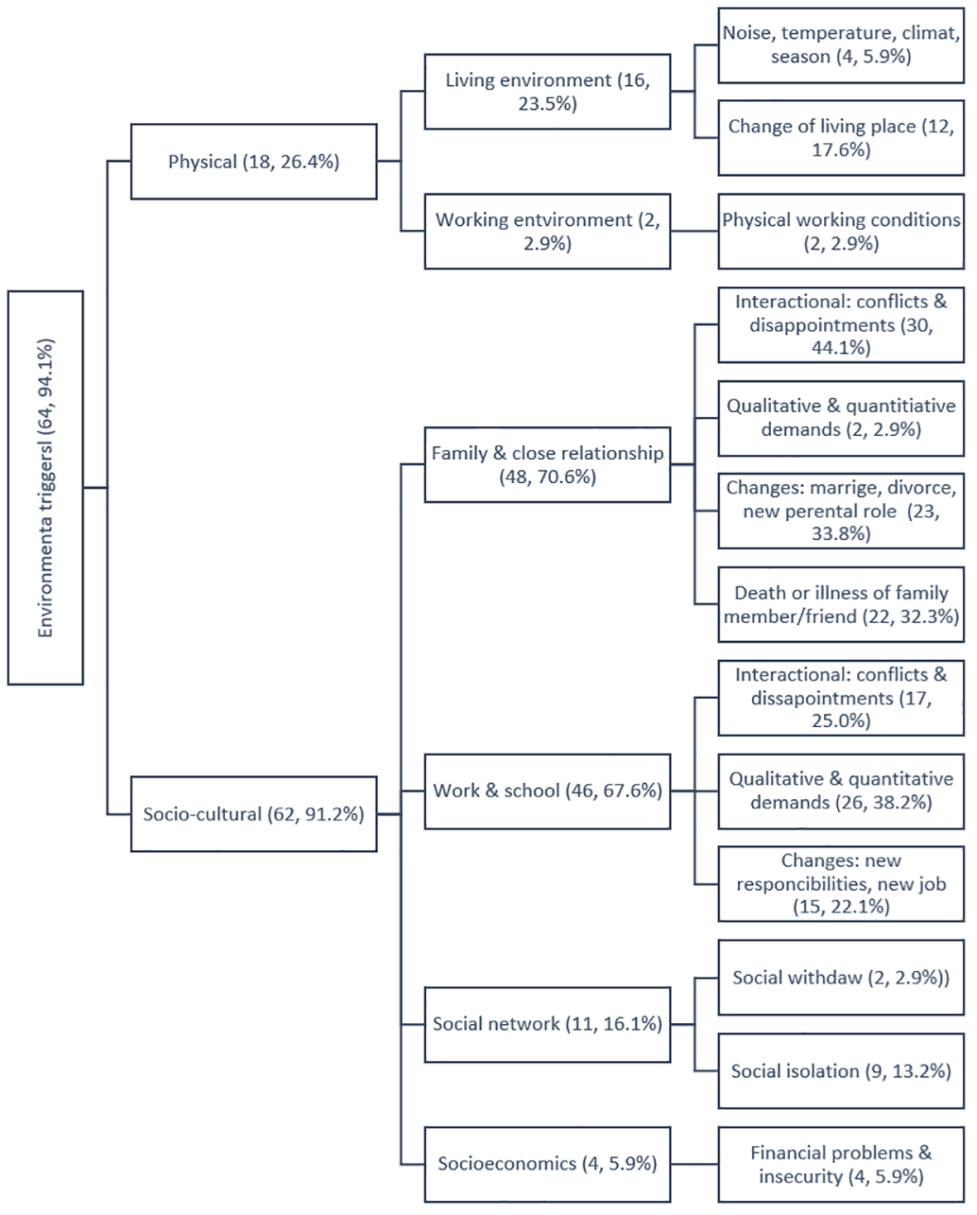
The three diagram of qualitative content analysis of environment triggers of depressive episode during the prodromal phase from the patient’s perspective (n, %).

**Figure 2:**
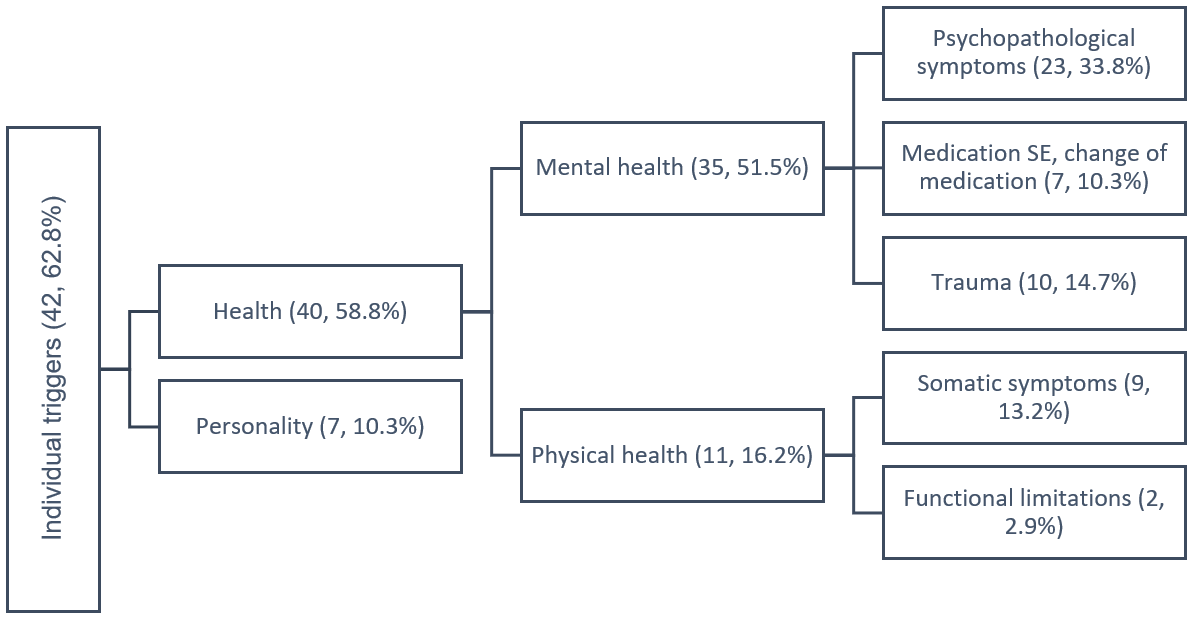
The tree diagram of qualitative content analysis of individual triggers of depressive episode during the CHR-D (Clinical High Risk for Depression) from the patient’s perpective (n, %).

All stressors were divided in two major groups relating to patients’ experience with CHR-D: (1) environmental stressors and (2) individual factors. Concept maps of the two themes’ code trees are presented in **Figures 1** and **2**, displaying both the themes and sub-themes, supplemented by frequencies of occurrence in our study population. Environmental stressors (**Figure 1**) were grouped deductively into physical environment and sociocultural environment. Individual stressors (**Figure 2**) were composed of health-related factors and personality-based sources. Below, all categories of stressors will be described in more details.

### Sociocultural environment

Four inductively derived themes for sociocultural environmental stressors were identified: (1) interpersonal stress in family and close relationships, (b) occupational stress from work and school, (2) (3) social network factors like withdrawal and social isolation, and (4) socioeconomic stress from financial difficulties and financial insecurity. We also identified four main characteristics of interpersonal stress factors. First, conflicts and disappointments were the leading interactional stress factor in close relationships. Second, high quantitative or qualitative demands in terms of high level of responsibility or complexities of the demands were reported. Thirdly, life changes such as marriage, childbirth, new parental roles, or divorce were reported as cause for depressive symptoms. Additionally, the fourth trigger category included the death or illness of family members.

Work related stressors (‘Work & School’) is another category of stressors that occur in work or educational settings. These stressors can be characterized by (1) interpersonal conflict or disappointment at work or school/study settings, (2) high levels of qualitative or quantitative demands at work or school/study settings, and (3) changes at the workplace such as new responsibilities, personnel or structural changes, or new employment.

As an example, detailed extracts of participant quotes across these more frequented two themes: (a) family and close relationships and (b) work and school, are displayed in **Table 5**.

**Table 5:** Participant quotations and themes: Social and cultural environment.

	Category	Extract of participants quotations
Family and close relationship	Interactional stress in family and close relationship: conflicts and disappointments	Relationship: partner very complicated, - ‘carrot & stick’. Conflict about the father’s inheritance with siblings Relationship problems: lack of attention from husband, poor recognition Feeling being misunderstood On-off relationship becomes more and more “broken”‘ Current partnership (only platonic) Relationship problems (not being able to talk to anyone) Disappointment: good friend suddenly broke off contact. without any explanation Relationship with “borderline” person; a lot of emotional back and forth Lack of support from the husband Relationship problems: jealousy and violence in close relationship
	Changes in family and close relationship	Decision made for wedding Planning of wedding Wedding-(stress) Changes after the birth of the second child Child given to day nursery for the first time Divorce Separation of the partner
	Death or illness of family member or close friend	Friend run over by truck while pilgrimage in Spain Death of husband Health problems of parents Mother diagnosed of brain tumour Brother very sick: kidney and pancreas cancer Death of father Suicide of brother-in-law Death of mother Father get seriously ill
Work and school	Interactional stress at work (school): conflicts and disappointments	Harassment/bullying at work Stress at work, felt abandoned by boss Stress at work with colleagues, mobbing Did not get expected permanent position at the university Problems with boss, dissatisfaction and anger Bossing Mobbing at the workplace New socially incompetent boss Teasing from the boss Poor recognition of work
	High qualitative or quantitative demands at work or in school	Increased work demands and self-demands Too much work. resignation Master’s thesis, totally immersed, only researched Overload at work. took on too much responsibility Pre-diploma, time before extremely stressful. hardly slept Completion of book manuscript No free time as a freelancer Graduation diploma–> stress Strong pressure to perform at university (self-imposed)
	Changes at work or school	New job as team leader. increased responsibility Employment contract was not extended 06/2008 Semester abroad, at first she was doing very well, towards the end doubts about… Change from study to job Difficulties in finding a job after graduation. starting a career in veterinary medicine New reform in work (technologization) New project at work

Social network stressors included social withdrawal or experience of poor social support or poor network. Socioeconomic stressors include financial difficulties or insecurity.

### Physical environment

Physical environment stressors can be divided into living and working environments (**Figure 1**). Both settings were reported to cause major depressive episode due to factors such as temperature, noise, or loss of living or working space.

### Individual factors

Individual factors that can serve as stressors are divided into (1) health factors and (2) personality. For mental health factors, patients reported stressors as psychopathological symptoms, changes in psychiatric medication, side effects of antidepressants, or reactivation of trauma. Physical factors such as somatic symptoms or functional limitations due to somatic disease were also mentioned as stressors caused development of depression. In terms of personality, higher emotional reactivity, perfectionism, and high self-demands are often reported as stressors for depressive episodes from the patient’s perspective.

### Quantitative descriptive analysis of stressors

The frequency analysis revealed the highest prevalence of environmental stressors group (94.1%) in comparison to individual stressors (62.8%) reported in this study sample of patients with depressive disorders. The most extensive subgroup of environmental stressors was the sociocultural environment with 91.2% of patients having mentioned this as a stressor caused the depressive episode, including subgroups of (1) family and close relationships, mentioned by 70.6% of patients; (2) work and school - 67.6%; (3) social network - 16.1% followed by (4) socioeconomics, 5.9%. Stressors at work or school were mentioned by 67.6% of patients. The most frequent reported environmental stressor in work or school settings was high quantitative or qualitative demands (38.2%), followed by interactional stressors including conflicts and disappointments (25%), and changes at the workplace (22.1%).

Significant gender differences were observed, with commonly reported health problems in women.

## Discussion

The current research provides a qualitative and quantitative exploration of precipitating stress-related factors from the patient’s perspective at the CHR-D. All patients reported at least one stressor that during the prodromal phase triggered the later (or index-) major depressive episode, the majority of them reported several cumulating stressors. Based on the groups of stressors described in the patient survey, we developed a model of the stressors that were identified in the prodromal phase as stressful factors before the onset of the disease.

Results demonstrates that patients with depressive disorder typically report multiple stressors, with the most common number being four. Category ‘Family & Close Relationships’ is consistently the most reported stressor across all levels, highlighting its significant impact on depressive episodes. Category ‘Work & Study’ impact remains a prominent stressor through the first three levels, suggesting its substantial role in the onset of depressive episodes. Psychopathology and ‘Somatic Health’ stressors are persistently relevant but less dominant compared to ‘Family & Close Relationships’ and ‘Work & Study’. Environmental Factors and Socio-economic are the least reported, suggesting they might be less significant triggers compared to others listed.

The relevance of each type of stressor generally diminishes as the number of stressors increases, indicating that primary stressors might have a stronger influence on triggering depressive episodes compared to secondary or additional stressors. The distribution of stressor sources varies as additional stressors are reported. ‘Family & Close relationships’ and ‘Work & Study’ were the most prominent initial stressors. As the number of reported stressors increases, the proportion of patients reporting these stressors decreases, while other types like psychopathology and somatic health maintain a steady but lower presence. Environmental factors and socio-economic stressors are consistently less reported across all levels.

Significant gender differences were found in the stressors related to ‘‘Family & Close Relationships’, ‘Work & Study’, and ‘Psychopathology’, with women reporting higher exposure to these factors compared to men.

In regards to single stressors analysis, interactional stress was found to play a crucial role in the development of a depressive episode from the patient’s perspective, with relationship and family stressors being the most frequently mentioned stressors in our study. The main stressor reported consists of interpersonal conflicts and disappointments in close relationship.

The results indicating that patients with depressive disorder typically report multiple stressors, with the most common number being four, can be explained by the multifaceted nature of depression. Depression often arises from a complex interplay of various stressors, rather than a single cause. This finding aligns with existing literature that highlights the cumulative effect of multiple stressors on mental health (31–34). The cumulative stress theory suggests that the accumulation of multiple stressors over time increases the likelihood of developing depressive symptoms. Each additional stressor can compound the individual’s overall stress burden, making it more difficult to cope and increasing the risk of depression (32). Stressors are often interconnected. For example, financial stress can lead to problems in relationships and increased work pressure, which in turn can contribute to feelings of inadequacy and hopelessness. This interconnection of stressors can create a feedback loop that exacerbates depressive symptoms (31, 33). Kendler et al. (33) explores the causal relationship between multiple stressful life events and the onset of major depression, highlighting the cumulative effect of stressors. Hammen (34) discussed how chronic and acute stressors are linked to the onset and course of depressive disorders, emphasizing the role of multiple stressors. Individuals with depressive disorder may have lower resilience to stress, making them more susceptible to the negative effects of multiple stressors. This can result in a heightened perception and reporting of stressors (34).

Remarkably, this result is surprising as this stressor has been well-known, but not as a prominent stressor preceding the outbreak of a manifest depression. Previous studies on family stress and depression have focused on the impact of family dynamics, relationships, and circumstances on individuals’ mental health, particularly examining how stress within the family can contribute to the development and exacerbation of depression. Research has shown that certain family environments, such as high levels of conflict, dysfunctional communication patterns, parental psychopathology, and emotional neglect, can contribute to increased stress levels within the family, heightening the risk of depression among family members. In details, one of the well-known concept of expressed emotion (EE) shows, that particularly criticism and emotional over-involvement, as well as marital distress, have been associated with a patient’s psychological outcome, however, robust results are only available for schizophrenia (35). Poor reciprocity in close social relationship was shown to be associated with poor health outcomes (36, 37). Several studies have demonstrated that marital distress pre-dates or leads to the occurrence of depression (33, 38). A 10-year prospective follow-up study confirms the decrease in marital satisfaction over time in the long-term course of depression (39). However, the results are controversial, as some studies have not found this association (40).

There is convincing evidence that poor social relationships negatively impact mental health (41). Life events were reported to play a central etiological role in the development of depression, with life events tied to changes involving loss (of relationship, role or sense of self), danger/threats (of the future role, conflicts in the core social roles, threats to plans you have made), or punishing environment (humiliation, entrapment) being most predictive (42).

The second stressor mentioned by patients related to CHR-D was high qualitative and quantitative demands at work. That is in the line with previous epidemiological research findings for work stress and risk of depression (20, 43, 44). Significant association was previously reported particularly between depression and adverse working conditions in terms of effort-reward imbalance (19), poor organizational justice (45), and job strain at the workplace (46).

Life changes within the close family circle and close friends were the third most frequently named stressor. Especially marriage, birth and separation were often experienced as stressors caused depression onset as junctures of increased decisions in these phases, which are referred to as “stress”. Life events as a risk factor for depression specifically has been subject of research investigation for centuries. Particularly, Monroe and Harkness (42) reported that approximately 70% of patients with first and 40% with recurrent episodes of depression are preceded by a severe stressful life event. Earlier, Brown and Harris (47) found that 80% of individuals with depression had a prior severe stressful life event. Growing literature on particularly on severe or negative life events and symptoms of depression have examined the effects of chronic stress on the onset and symptomatic presentation of MDD (34). Such negative stressful life events such as the death of a loved one, divorce, job loss, or financial troubles were previously reported to be major stressors for depression (42, 47). Remarkably, although some of these transitions are perceived as positive (e.g., marrige, giving birth), they received a high ranking in our study sample of patients with MD. In accordance with our findings on this positive and negative life events, Moustafa et al. (48) conducted a systematic literature review of the life transition factors underlying the occurrence of depression following major life transition and provided a theoretical framework that explains depression caused by transitions in women. The review shows that major common life transitions can cause depression if they are sudden, major, and lead to loss or change of life roles. One of the possible explanations within the theoretical frame was the capability to accept the new role (e.g., accepting new role as a mother) or find novel life roles instead (e.g. after children leave family home or after divorce) (48).

The observed gender differences in stressors related to family and close relationships, work and study, and psychopathology can be attributed to several factors. First, women often juggle multiple roles, such as caregiver, professional, and household manager, which can lead to higher stress levels in both family and work domains (49). Second, women may be more emotionally responsive and sensitive to interpersonal stressors, which can result in higher reported stress in family and close relationships (50). Third, women may face more workplace stress due to gender discrimination, unequal pay, and balancing career and family responsibilities (51).

### Stress measurement in the CHR-D

Based on the qualitative material obtained from the interviews, the precursors would need to be classified according to a set of analytical categories that relate to psychosocial theories of depressive disorders. Particularly, all events related to the social environment could be classified into one of the following three types: (1) physical environment, (2) sociocultural environment, and (3) individual stressors. Inductively, stressors related to the social and cultural environment could be classified into four theoretical subtypes: (a) interpersonal: family and close relationships; (b) occupational: work and school; (c) social network: withdrawal and isolation; and (d) socioeconomics. Additionally, the following characteristics of stressors could be identified: (a) interactional stress: conflicts and disappointments; (b) high qualitative and quantitative demands; (c) changes; and (d) death or illness of family member. Relationship and family problems were the most frequent stressors reported, followed by work stressors and health disturbances. These is suggested preliminary model underlying the construction of a standardized assessment of precursors of prodromal symptoms in patients suffering from depressive disorders to be used as a first step for the development of the new screening instrument for the identification of the clinical high risk state of depression.

Brown and Harris (47), in one of the still most comprehensive studies aiming to capture systematically all stressors related to depression, found a particular type of chronic stress, which they called “major difficulties,” to be associated with the onset of MDD in a community sample of women. These stressors were defined as ongoing stressful conditions that are highly unpleasant, threatening to an individual’s plans, goals, and aspirations for the future, and present for a minimum of two years. Some years earlier, the social readjustment model of Holmes and Rahe (52) provided a method of life stress quantification and established fixed parameters to rate and compare 43 severe life stressors. However, it is not clear, which factors have a higher predictive validity for the development of depression.

Later on, King et al. (53) have developed a first risk algorithm including recognized risk factors for major depression over 12 months in 5216 general practice attendees in Europe and validated its use in 1732 attendees in Chile. The aim of this study was to determine the key factors in a valid clinical prediction algorithm. 39 known risk factors to construct a risk model for onset of major depression were measured.

Five risk factors in the final model were immutable (age, sex, educational level achieved, results of lifetime screen for depression, and family history of depression) and 4 were mutable factors relating to current status (Short Form 12 physical health and mental health subscale scores, unsupported difficulties in paid and/or unpaid work, and experiences of discrimination). In the study presented here, as a first step, a comprehensive preliminary mapping of all sources of stress relevant to MD, based on a patient survey was developed. Further investigations are needed to proof this model longitudinally on the larger study sample using a control group of healthy adults.

### Limitations

Some limitations should be taken into account. First, findings are based on a relatively small study sample and retrospective identification of prodrome and its stressors. To reduce the recall bias, only partly remitted and remitted patients were included in the study. Second, individual variability may question a shared criterion to evaluate and compare stressors. In our study design were not able to differentiate stressors from vulnerabilities. While the last point has been systematically controlled (54), different research methodologies have dealt with challenges of individual variability and cognitive appraisals. Moreover, researchers are aware that certain environmental risks are stable social or cultural conditions. Epps and Jackson (55) summarize risk factors as stable conditions in four domains: family, socio-political, cultural and economic contexts. Third, we didn’t classify perceived stress according to it severity. In earlier investigations, it has been argued that severe life events are the most significant causal factor for depression (47). However, the current study focused on the patient’s perception of stress that might be relevant to depression development. In detail, free report of stressful life events before onset of the prodromal phase in regard of their role in the development of depression from the patient’s perspective was obtained. While the current study might not have addressed the concurrent experience of multiple stressors, recognizing this limitation provides a valuable direction for enhancing the depth and applicability of future depression research. This approach would more accurately reflect the real-life complexities of how stressors interplay and impact mental health. Further limitation of our study was the potential ambiguity in distinguishing between stressors explicitly mentioned by participants and those inferred through qualitative analysis. However cross-coding by two researchers with regular consensus meetings improve accuracy and consistence of the coding. Moreover, incorporating detailed gender analysis in future studies could enhance understanding and contribute to more personalized, gender-sensitive approaches in depression prevention and treatment. Finally, the findings of this study are restricted to a descriptive level, thus limiting generalization.

### Strengths

These limitations are based on several strengths of the study. To our knowledge, this is the first study evaluated a set of stressors of a prodromal phase for depression through clinical interview and comprehensive exploration of all possible stressors. The current study addresses this gap by analysing free-text responses from a feasibility study on depression. An analysis of patient’s own words permits a nuanced up-dated examination of the specific challenges and life experiences regarding psychosocial stressors and their impact on depression development.

Including less frequently mentioned themes, such as physical environmental stressors, is essential for a comprehensive understanding of factors influencing the prodromal phase of depression. This holistic approach ensures the study acknowledges the complex and multifactorial nature of depression, capturing a wide spectre of stressors. It prevents overlooking potential contributors that may be critical for specific subgroups. Comprehensive data can lead to more effective, personalised intervention strategies. Including these themes enriches the study’s findings, enhancing the predictive power of models and the effectiveness of prevention strategies, ensuring adaptability to diverse individual circumstances and environmental contexts.

Previous epidemiological investigations on the stressors that trigger depression focused on the precipitating factors and not on the characteristics of individuals or course of disease. Moreover, this is the first study identifying subtypes of triggering stressors and its dependence from course of disease.

Studying stressors prior to the onset of a major depressive episode is crucial for gaining a deeper understanding of the association between stress and depression. Identifying these stressors can help in developing more effective measures to reduce the risk of depression. Our study, conducted as part of a feasibility study, aims to provide valuable insights into the early signs of depression and contribute to the identification of psychosocial predictors in the prodromal stage of depression. Including this investigation of stressors in the future longitudinal studies might improve predictive models. Further investigating who is more vulnerable to these types of stressors could enhance future prediction. This investigation could lead to new inputs to improve risk stratification.

In terms of public health policy implications, two conclusions can be drawn from our findings. All patients with affective disorders have reported stressors before the onset of prodromal symptoms; gender differences were observed. Identification of a set of triggers at the early stage of depressive disorder may increase opportunities for early specific intervention. Through data interpretation, the results of the qualitative content analysis can support the development of new instruments for risk assessment of depression and providing detailed descriptions of particular phenomena.

## Conclusions

Extending previous studies in non-clinical samples, this study presents an initial exploration into the specific stressors characteristic of the prodromal phase of major depressive episodes (MDE). The identification of distinct stressors during the prodromal phase offers promising directions for early intervention and preventive measures. However, these findings must be viewed as preliminary until validated by further studies with larger and more diverse populations.

The clinical significance of our research, while encouraging, should be interpreted with restraint. We emphasize the need for additional research to confirm these stressors’ roles and to refine the tools necessary for early detection and intervention in clinical settings. Moving forward, replication of these results, along with extended longitudinal studies, will be crucial to substantiate the clinical applicability of our findings and to support the development of interventions that can effectively mitigate the progression to onset depression.

## Outlook and implications for practice

Identification of a set of stressors at the early stage of depressive disorder may increase opportunities for early specific intervention. Through data interpretation, the results of our content analysis can support the development of new instruments for risk assessment of depression and providing detailed descriptions of particular phenomena. A development of stressors and triggers checklists that precede depression should be a next step in prediction research. Data derived from the current study provides helpful information to develop a new instrument and validate it in a prospective study design.

Thus, it is crucial for early recognition and prevention of depression to identify those stressors and indicate that resources for psychosocial support should be addressed by prevention.

Future studies should examine stressors of the prodrome detected in this investigation to capture possible categories specific for depression development. The final goal is the development of valid, reliable as well as economical clinical and multimodal instruments (clinical interviews and self-rating questionnaires) for the early detection of MDE in various settings with special conceptual focus on the lifespan, including the transition phase of early adulthood as well as older age. This developmental perspective is already well advanced in the field of psychoses (56, 57) and encourages the transfer to the to the field of affective disorders.

## Data availability statement

The raw data supporting the conclusions of this article will be made available by the authors, without undue reservation.

## Ethics statement

The studies involving humans were approved by Ethics Committee of the Ludwig-Maximilians University. The studies were conducted in accordance with the local legislation and institutional requirements. The participants provided their written informed consent to participate in this study.

## Author contributions

EM: Conceptualization, Data curation, Formal Analysis, Funding acquisition, Investigation, Methodology, Project administration, Resources, Supervision, Validation, Visualization, Writing – original draft, Writing – review & editing. GS: Conceptualization, Investigation, Resources, Supervision, Writing – original draft, Writing – review & editing. EG: Conceptualization, Investigation, Methodology, Resources, Supervision, Validation, Visualization, Writing – original draft, Writing – review & editing. VS: Conceptualization, Data curation, Formal Analysis, Investigation, Methodology, Software, Visualization, Writing – original draft, Writing – review & editing. CK: Conceptualization, Formal Analysis, Investigation, Methodology, Resources, Software, Validation, Visualization, Writing – original draft, Writing – review & editing. UD: Conceptualization, Methodology, Resources, Supervision, Validation, Visualization, Writing – original draft, Writing – review & editing. TH: Conceptualization, Data curation, Formal Analysis, Methodology, Resources, Software, Supervision, Validation, Writing – original draft, Writing – review & editing. GR: Conceptualization, Methodology, Project administration, Resources, Supervision, Validation, Writing – original draft, Writing – review & editing. MR: Conceptualization, Methodology, Resources, Supervision, Validation, Writing – original draft, Writing – review & editing. LD: Conceptualization, Investigation, Methodology, Project administration, Resources, Supervision, Validation, Visualization, Writing – original draft, Writing – review & editing. CT: Data curation, Formal Analysis, Methodology, Validation, Writing – original draft, Writing – review & editing. MK: Conceptualization, Methodology, Validation, Supervision, Writing – original draft, Writing – review & editing. SR: Conceptualization, Methodology, Supervision, Validation, Writing – original draft, Writing – review & editing. AF: Conceptualization, Investigation, Methodology, Resources, Supervision, Validation, Writing – original draft, Writing – review & editing. FS: Conceptualization, Methodology, Resources, Supervision, Validation, Visualization, Writing – original draft, Writing – review & editing. NW: Conceptualization, Data curation, Formal Analysis, Investigation, Methodology, Supervision, Validation, Visualization, Writing – original draft, Writing – review & editing.
